# Subclinical vestibular dysfunction in migraine patients: a preliminary study of ocular and rectified cervical vestibular evoked myogenic potentials

**DOI:** 10.1186/s10194-015-0578-5

**Published:** 2015-11-02

**Authors:** Chul-Ho Kim, Min-Uk Jang, Hui-Chul Choi, Jong-Hee Sohn

**Affiliations:** Department of Neurology, Chuncheon Sacred Heart Hospital, Hallym University College of Medicine, 153 Gyo-dong, Chuncheon-si, Gangwon-do 200-704 Republic of Korea

**Keywords:** Vestibular evoked myogenic potential (VEMP), Ocular VEMP, Cervical VEMP, Migraine

## Abstract

**Background:**

Many studies have identified various vestibular symptoms and laboratory abnormalities in migraineurs. Although the vestibular tests may be abnormal, the changes may exist without vestibular symptoms. To date, vestibular-evoked myogenic potential (VEMP) has been the easiest and simplest test for measuring vestibular function in clinical practice. Cervical VEMP (cVEMP) represents a vestibulo-collic reflex, whereas ocular VEMP (oVEMP) reflects a vestibulo-ocular pathway. Therefore, we determined whether ocular and rectified cervical VEMPs differed in patients with migraine or tension type headache (TTH) and compared the results to controls with no accompanying vestibular symptoms.

**Methods:**

The present study included 38 females with migraine without aura, 30 with episodic TTH, and 50 healthy controls without vestibular symptoms. oVEMP and cVEMP using a blood pressure manometer were recorded during a headache-free period. From the VEMP graphs, latency and amplitude parameters were analyzed, especially following EMG rectification in cVEMP.

**Results:**

With respect to oVEMP, the migraine group exhibited significantly longer mean latencies of bilateral n1 and left p1 than the other groups (*p* < 0.05). Amplitudes of n1-p1 were lower than in other groups, but the difference did not reach statistical significance. In regards to cVEMP, p13 and n23 latencies and amplitudes after rectification did not differ significantly among groups.

**Conclusions:**

An abnormal interictal oVEMP profile was associated with subclinical vestibular dysfunction in migraineurs, suggesting pathology within the vestibulo-ocular reflex. oVEMP is a more reliable measure than cVEMP to evaluate vestibular function in migraineurs, although results from the two tests in patients with migraine are complementary.

## Background

Dizziness and vertigo are frequent symptoms accompanying primary headache disorders, especially migraine [1]. Migraine has long been associated with various vestibular symptoms and several vestibular syndromes [2]. Additionally, several studies have identified several vestibular laboratory abnormalities in migraineurs [3].

Of the various methods used to evaluate the vestibular system, vestibular evoked myogenic potential (VEMP) is a non-invasive and simple clinical test. Cervical VEMP (cVEMP) represents an uncrossed vestibulo-collic reflex, which assesses saccular function, the inferior vestibular nerve and vestibular nuclei, and serves as a pathway through the lower brainstem to the motor neurons of the sternocleidomastoid muscle [4]. The more recently described ocular VEMP (oVEMP), a manifestation of a crossed vestibulo-ocular pathway, reflects predominantly utricular function and involves the medial longitudinal fasciculus, oculomotor nuclei and nerves, and extraocular muscles following activation of the vestibular nerve and nucleus [4, 5]. While cVEMP descends via the vestibulospinal tract through the lower brainstem, oVEMP ascends via the medial longitudinal fasciculus through the upper brainstem. Additionally, recent studies suggest that oVEMP is produced by otolith afferents in the superior vestibular nerve division, whereas cVEMP, evoked by sound, is believed to be an inferior vestibular nerve reflex [6]. Using oVEMP and cVEMP together allows for the evaluation of both ascending and descending vestibular pathways in the brainstem and identifies a higher percentage of abnormalities [4]. Thus, the combined measures of oVEMP and cVEMP provide complementary information.

VEMP presentation differs in individual patients according to the method used for assessment, diagnosis of migraine subtype, and the presence of vestibular symptoms, as reported in literature. Several authors have reported absent or delayed cVEMPs [7–10], whereas others have found cVEMPs of normal latency but reduced amplitude in migraineurs [11, 12]. In contrast with most previous studies, a normal interictal cVEMP profile was reported in patients with migraine with or without aura and vestibular migraine [13]. Recently, interest in oVEMP studies for migraine has increased. High rates of absent oVEMP and higher amplitude asymmetry ratios or reduced amplitudes have been shown in vestibular migraine (VM) [14, 15], whereas prolonged latency and lower amplitudes were found in migraineurs without vestibular symptoms [16].

Although previous VEMP reports have been inconsistent, VEMP remains the easiest and simplest method for measuring vestibular activity in clinical practice to date. Measurement of both ocular and cervical VEMPs provides more information because the results are complementary. Additionally, several studies on patients with migraine without vestibular symptoms have reported vestibular deficits in various vestibular function tests. In particular, findings such as defective oculomotor function, dysfunctional equilibrium, and peripheral and central vestibular deficits have been described [17–21]. Patients with tension-type headache (TTH) often report balance disorders or subjective imbalance [22, 23]. However, little is known about vestibular function in those with TTH without manifested vestibulopathy.

Thus, we hypothesized that migraineurs with no accompanying vestibular symptoms exhibit subclinical vestibular dysfunction. We investigated vestibular function using ocular and rectified cervical VEMP methods in patients with migraine without aura and those with episodic TTH during headache-free periods.

## Methods

### Subjects

This study collected data obtained from consecutive first-visit patients with migraine without aura and episodic TTH treated in the neurology outpatient department of a university hospital. All participants were between 20 and 60 years of age, and only females were included to eliminate age and gender bias [24–26]. Headache diagnoses were classified by a board-certified neurologist based on the criteria of the International Classification of Headache Disorders-3 beta version (ICHD-3β) using patient history, a neurological examination, and laboratory or neuroimaging studies. To exclude other primary headaches, patients were required to have at least a 1-year history of migraine or TTH headaches prior to enrollment. Patients who had auras or vestibular symptoms during headache attacks were excluded. In total, 38 patients with migraine without aura and 30 patients with episodic TTH based on the ICHD-3β were enrolled in the study. Subjects with episodic TTH were defined as those with headaches lasting from 1 to 15 days per month (frequent episodic TTH). The control group consisted of age-matched volunteers. We recruited the control group by inviting persons who accompanied the patients to join the study (e.g., friends) and also through advertisements (e.g., posted notices in the hospital). Controls were free of headaches for at least three months prior to the study, experienced no more than an occasional mild headache (<5 times per year) and had not sought medical treatment for headaches.

All participants were underwent physical and neurological examinations performed by an experienced neurologist. Participants were asked to complete a questionnaire regarding their headache symptoms, including frequency, duration, and intensity, during the previous 4 weeks. Headache frequency (days/week) was calculated by dividing the number of days with headaches by 4 weeks. Headache duration (hours/day) was calculated by dividing the sum of the total hours of headaches by the number of days with headaches and headache intensity (numeric rating scale [NRS]: 0 = no pain to 10 = unbearable pain) was calculated as the mean NRS for days with headaches. We also obtained a comprehensive neuro-otological history from all participants. The detailed interview for assessing vestibular symptoms in headache patients or diagnosing VM according to the ICHD-3β included questions about clinical features (e.g., main type of vertigo and duration, frequency, severity) and concomitant symptoms. Exclusion criteria included subjects with hearing loss, middle ear disease or surgery, history of vestibular disease, history of recurrent vertigo or vertigo that lasted more than one day or required hospitalization, a cervical disorder that affected head movement, the presence of neurological disorders (e.g. stroke, multiple sclerosis), pregnancy, daily medication to prevent headaches and/or antidepressant medication, medication-overuse headache, and patients with VM.

Written informed consent was obtained from all subjects prior to enrollment. The university hospital ethics committee approved this study.

### VEMP recordings

VEMP tests were performed by an examiner blinded to group and patient clinical examination data. VEMP recordings were performed using a Nicolet EDX EMP/EP machine (Natus Neurology, Middleton, WI, USA). Patients with headache underwent VEMP testing on headache-free days. Specifically, recordings in migraine patients were obtained interictally at least 3 days after the last and before the next migraine attack.

### oVEMP

For oVEMP testing, the active electrode was placed ~1 cm below the center of the inferior eyelid contralateral to the sound stimulation, with the reference electrode located 15 mm below the active electrode and the ground electrode on the forehead. Patients were tested in a seated position. During the test, patients were asked to look upward to a fixed point 2 m away and 25-30° above the horizontal line. Electromyography (EMG) signals were amplified and band pass-filtered between 30 and 3000 Hz. Sound stimuli were presented through headphones as short tone burst sounds (500 Hz) at a frequency of 5 Hz. In total, 100 stimuli were applied to each ear and repeated twice consecutively at 130 dB normal hearing level (nHL).

### cVEMP

For cVEMP testing, the active electrode was placed on the upper one-third of the sternocleidomastoid (SCM) muscle, ipsilateral to the sound stimulation, with the reference electrode on the anterior margin of the clavicle and the ground electrode on the forehead. Patients were tested in a seated position. To contract the SCM, we used the blood pressure cuff method [27]. Subjects had to flex the head ~30° forward and rotate it ~30° to the opposite side. While holding the cuff between the right hand and jaw, the subject pushed with her head against the hand-held cuff to generate a cuff pressure of 40 mmHg. The obtained cuff pressures and background muscle activity based on visual feedback system of the VEMP machine were monitored by the subject and investigator during the recording period. EMG signals were amplified and band pass-filtered between 20 and 2000 Hz. Sound stimuli (500 Hz) were presented through headphones as rarefaction clicks 0.1 ms in duration and at a frequency of 5 Hz. In total, 128 stimuli were applied to each ear and repeated twice consecutively at a 125 dB nHL.

### VEMP analysis

From the oVEMP graphs, unrectified signals from 100 trials were averaged. The first negative and positive responses were designated as n1 and p1 waves, respectively. The oVEMP response was only considered reliable if the n1 and p1 peaks were reproducible in two consecutive trace runs. Additionally, the cVEMP response was only considered reliable if the p13 and n23 peaks were reproducible in two consecutive runs of the unrectified trace. The p13-n23 responses were observed best in the unrectified trace. Initial positive and negative polarities of the waveform with peaks were termed p13 and n23 on the basis of their respective latencies. Rectified values were used since the VEMP response amplitude is significantly affected by the force of muscular contraction or stimulus intensity. After rectification (Synergy Reader software, version 20.1), peak latencies of p13 and n23 and amplitude parameters p13 and n23 were measured. The results of both runs were averaged, providing the final response from which the peak-to-peak amplitude (n1-p1) and absolute latencies (n1, p1) in oVEMP and rectified amplitude and absolute latencies (p13, n23) in cVEMP were derived. Interside differences of n1 and p1 latencies in oVEMP and p13 and n23 latencies in cVEMP were calculated. Amplitude asymmetry ratio (AR) was calculated in oVEMP and cVEMP as follows: (larger response - smaller response) / (larger response + smaller response) × 100 [4].

### Statistical analyses

Statistical analyses were performed using ‘R’ (version 3.01; R Foundation for Statistical Computing, Vienna, Austria) and P-values <0.05 were considered to indicate statistical significance. The planned sample of 38 migraineurs and 46 healthy subjects resulted in a power of 90 % for detecting a 40 % reduction in the bilateral oVEMP response at a significance level of 0.05 using a two-sided Fisher’s exact test [16]. Additionally, the sample size calculation for the t-test to detect the difference in N1 latencies between the migraine and healthy control groups required 24 and 48 subjects, respectively. Data were expressed as the means ± standard deviation (SD) for continuous variables and as numbers (rates) for categorical variables. Continuous variables were compared using a two-sample *t*-test or Wilcoxon’s rank sum test, whereas categorical variables were evaluated using the χ^2^ test or Fisher’s exact test. Results of oVEMP and cVEMP parameters were compared among three subgroups. Multiple group analyses were performed using one-way analysis of variance (ANOVA) or the Kruskal-Wallis test. Pair-wise comparisons were assessed using the Wilcoxon’s rank sum test with Bonferroni correction.

## Results

### Clinical characteristics

The present study included 38 females with migraine without aura, 30 episodic TTH and 50 healthy controls. Mean age in the migraine, TTH and control groups was 35.5, 33.3 and 35.1 years, respectively. The mean age did not differ significantly among groups. Clinical and headache characteristics are shown in Table 1.

**Table 1:** Clinical and headache characteristics of the study groups

	TTH (*n* = 30)	Migraine (*n* = 38)	Control (*n* = 50)	P-value
Age (years)	33.37 ± 13.70	35.58 ± 12.26	35.14 ± 13.47	NS
Frequency (day/week)	1.52 ± 0.93	2.14 ± 2.05	-	
Duration (hours/day)	5.35 ± 6.01	9.51 ± 8.83	-	
Intensity (NRS)	4.13 ± 1.61	6.29 ± 2.10	-	

Values are expressed as mean ± standard deviation

*TTH* tension-type headache, *NRS* numeric rating scale, *NS* non-significant

### oVEMP abnormalities

**Fig. 1:**
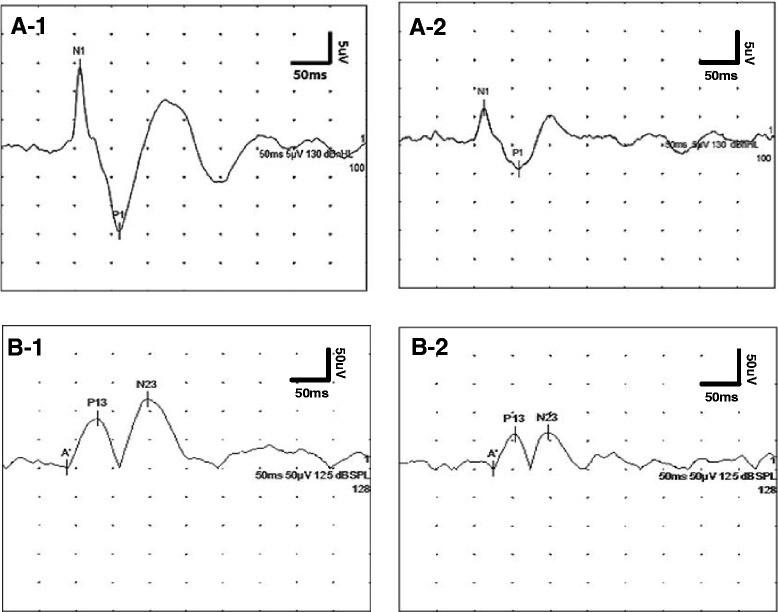
oVEMP and rectified cVEMP responses in normal subjects (**a-1**, **b-1**, respectively) and migraine patients (**a-2**, **b-2**, respectively). oVEMP: ocular vestibular evoked myogenic potential; cVEMP: cervical vestibular evoked myogenic potential

**Fig. 2:**
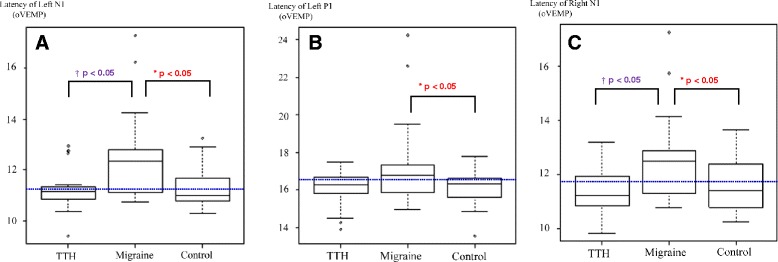
Box plots of latency of oVEMP. Latencies of **a** left n1, **b** left p1, and **c** right n1. Horizontal dashed lines indicate the grand average. *p < 0.05, statistically significant difference between migraine patients and healthy controls; † *p* < 0.05, statistically significant difference between migraine patients and patients with TTH; oVEMP: ocular vestibular evoked myogenic potential

Eight patients in the migraine group (7, 18.4 % unilateral; 1, 2.6 % bilateral) demonstrated absent oVEMP responses, while responses could not be obtained for three patients in the TTH group (2, 6.7 % unilateral; 1, 3.3 % bilateral) and five patients in the control group (3, 6.0 % unilateral; 2, 4.0 % bilateral). A low response rate was observed in migraineurs, but no statistical difference was detected in the response rate of oVEMP among groups (Table 2). In oVEMP, the migraine group had mean latencies of bilateral n1 and left p1 significantly longer than the other groups (*p* < 0.05). Mean amplitudes of n1-p1 were lower than in the other groups, but the difference did not reach statistical significance (Table 3). No significant difference was observed in the AR amplitude or interaural latency differences of oVEMP. Illustrated examples of oVEMP tracings in controls and patients with migraine are shown in Fig. 1. Box plots of statistically significant oVEMP parameters are shown in Fig. 2.

**Table 2:** VEMP response rates in headache patients and healthy controls

	TTH (*n* = 30)	Migraine (*n* = 38)	Controls (*n* = 50)
oVEMP			
Bilateral response, n (%)	27 (90.0 %)	30 (78.9 %)	45 (90.0 %)
Unilateral response, n (%)	2 (6.7 %)	7 (18.4 %)	3 (6.0 %)
No response, n (%)	1 (3.3 %)	1 (2.6 %)	2 (4.0 %)
cVEMP			
Bilateral response, n (%)	23 (76.7 %)	34 (89.5 %)	46 (92.0 %)
Unilateral response, n (%)	7 (23.3 %)	4 (10.5 %)	3 (6.0 %)
No response, n (%)	0 (0 %)	0 (0 %)	1 (2.0 %)

*TTH* tension-type headache, *oVEMP* ocular vestibular evoked myogenic potential, *cVEMP* cervical vestibular evoked myogenic potential

**Table 3:** oVEMP results of headache patients and healthy controls

Parameters	TTH	Migraine	Controls
Left side			
latency n1 (ms)	11.29 ± 0.78**	12.34 ± 1.43*,**	11.29 ± 0.73*
latency p1 (ms)	16.09 ± 0.83	17.05 ± 1.95*	16.11 ± 0.82*
n1-p1 interpeak latency (ms)	4.79 ± 0.87	4.75 ± 1.22	4.82 ± 0.92
n1-p1 amplitude	11.64 ± 6.73	8.00 ± 5.21+	11.66 ± 9.14
Right side			
latency n1 (ms)	11.42 ± 0.86**	12.41 ± 1.41*,**	11.58 ± 0.90*
latency p1 (ms)	16.41 ± 0.89	16.99 ± 2.02	16.20 ± 1.04
n1-p1 interpeak latency (ms)	4.99 ± 1.02	4.58 ± 1.24	4.62 ± 1.00
n1-p1 amplitude	11.29 ± 6.77	7.51 ± 4.47+	11.76 ± 10.79
Interside difference			
interaural latency diff., n1	0.64 ± 0.86	0.91 ± 0.97	0.74 ± 0.72
interaural latency diff., p1	0.64 ± 0.55	0.84 ± 0.95	0.78 ± 0.62
amp. asymmetry ratio, n1	0.23 ± 0.17	0.28 ± 0.17	0.23 ± 0.16
amp. asymmetry ratio, n1	0.22 ± 0.19	0.28 ± 0.21	0.24 ± 0.15

*p < 0.05, statistically significant between patients with migraine and controls; **p < 0.05, statistically significant between patients with migraine and patients with TTH; values are expressed as the means ± standard deviation

*TTH* tension-type headache, *oVEMP* ocular vestibular-evoked myogenic potential, *amp* amplitude

### cVEMP abnormalities

Four patients in the migraine group (4, 10.5 % unilateral; 0, 0 % bilateral), seven patients in the TTH group (7, 23.3 % unilateral; 0, 0 % bilateral) and four patients (3, 6.0 % unilateral; 1, 2.0 % bilateral) in the control group showed absent cVEMP responses. There was no statistically significant difference among the groups with respect to cVEMP response rate (Table 2). Illustrated examples of rectified cVEMP tracings in controls and patients with migraine are shown in Fig. 1. Additionally, p13 and n23 latencies and rectified amplitudes of cVEMP in migraine without aura and TTH patients did not differ significantly from those of healthy controls (Table 4). Moreover, no significant difference was observed in the amplitude AR or interaural latency differences of cVEMP.

**Table 4:** Rectified cVEMP results of headache patients and controls

Parameters	TTH	Migraine	Controls	*P* value
Left side				
latency p13 (ms)	12.78 ± 1.37	13.57 ± 2.37	12.84 ± 1.81	NS
latency n23 (ms)	20.82 ± 2.01	21.98 ± 3.01	21.32 ± 2.06	NS
p13 rectified amp (μV)	42.43 ± 19.10	38.61 ± 24.95	39.41 ± 21.81	NS
n23 rectified amp (μV)	53.22 ± 13.90	58.52 ± 37.73	50.95 ± 34.25	NS
Right side				
latency p13 (ms)	12.89 ± 2.45	13.68 ± 2.16	13.01 ± 2.00	NS
latency n23 (ms)	21.06 ± 2.17	22.09 ± 3.10	21.56 ± 2.29	NS
p13 rectified amp (μV)	38.97 ± 21.05	34.49 ± 23.40	41.79 ± 25.55	NS
n23 rectified amp (μV)	49.98 ± 15.70	46.38 ± 28.37	57.79 ± 39.72	NS

Values are expressed as the means ± standard deviation

*TTH* tension-type headache, *cVEMP* cervical vestibular evoked myogenic potential, *amp* amplitude, *NS* non-significant

## Discussion

In our study, significantly prolonged latency in oVEMP was detected in migraine without aura versus TTH and control groups. However, there was no significant difference in cVEMP parameters among the migraine, TTH and control groups. These results suggest pathology within the oVEMP pathway or the ascending utriculo-ocular reflex in migraineurs. Thus, migraineurs showed subclinical vestibulopathies with oVEMP abnormalities during a headache-free period.

cVEMP and oVEMP provide valuable information regarding the location and nature of the lesion(s) affecting central vestibular pathways because the vestibulo-collic and vestibulo-ocular reflex pathways diverge beyond the nerve root entry zone [4, 28]. Using oVEMP and cVEMP together allows for the evaluation of both ascending and descending vestibular pathways, resulting in the identification of a higher percentage of abnormalities [4, 28–30]. Patients who have brainstem involvement in multiple sclerosis or internuclear ophthalmoplegia show higher abnormality rates in oVEMP than in cVEMP [29, 31]. Additionally, oVEMP is more sensitive than cVEMP for detecting silent brainstem lesions in multiple sclerosis patients and vestibular dysfunction in VM patients [14, 29]. Furthermore, because oVEMP latencies are dependent primarily on afferent and efferent reflex limbs and central transmission, prolonged latencies are likely due to the degradation of central vestibular processing of otolith signals rather than a decline in peripheral vestibular function [26]. VEMP amplitudes can be used as independent quantitative measures of otolith function [4]. Thus, peripheral vestibular disorders frequently involve an absence of oVEMPs or decreased amplitudes, whereas prolonged latencies may indicate central vestibular lesions [32]. Significantly prolonged oVEMP latencies in our study suggest an underlying functional abnormality in the central vestibular system.

Herein, bilaterally or unilaterally absent oVEMP responses were observed in 21 % of patients in the migraine group, while absent cVEMP responses were found in 10.5 % of patients in the migraine group. Several previous reports showed absent oVEMP responses in the migraine group (53.3 %) and VM group (28 %), whereas absent cVEMP responses were detected in 8 % of VM patients [14, 16]. Similar to previous studies, a high unresponsive rate of oVEMP in migraineurs was observed in our study, although the difference did not reach statistical significance. These findings also suggest defective oVEMP pathways in migraineurs.

Various vestibular function test studies have been conducted on patients with migraines during the interictal period. Several works reported vestibular abnormalities in the form of involvement of peripheral or central vestibular pathways or both [18, 19, 33]. One study reported dysfunction in the vestibulo-ocular reflex, whereas another indicated underlying dysfunction in the vestibulospinal system [21, 34]. Other reports showed interictal dysfunction of vestibulocerebellar origin in migraineurs [20, 35]. These findings suggest that migraineurs without vestibular symptoms exhibit vestibular abnormalities, generally indicating subclinical vestibulopathies in patients with migraines. Additionally, the distribution between central and peripheral vestibular findings did not differ between VM and migraine patients [18]. More recently, in a report evaluating cVEMP and oVEMP pathways in patients with VM, the rates of abnormal oVEMPs were significantly higher without cVEMP abnormalities, similar to our study, although the subjects suffered symptoms on the same day of testing [14]. Thus, subclinical vestibular dysfunction may be an integral part of migraine pathophysiology and could be related to fundamental pathophysiological similarities between migraine and VM. Recently, positron emission tomography (PET) studies have demonstrated thalamo-cortical involvement or increased thalamic activation in VM patients [36, 37]. Additionally, voxel-based morphometry studies have identified gray matter volume reductions in patients with VM [38]. These functional and structural alterations in patients with VM resemble those previously described in patients with migraine. VM likely represents the pathophysiological paradigm of a connection between migraine and the vestibular system [39].

Subclinical vestibulopathy in migraineurs may be related to multiple potential interactions between the trigeminal and vestibular systems at various levels. In migraine patients, stimulation of the trigeminal nuclei has produced spontaneous nystagmus [40]. Conversely, vestibular nuclei receive both serotonergic inputs from the dorsal raphe nucleus and noradrenergic inputs from the locus coeruleus, and activation of these pain structures during migraine can affect central vestibular processing [3]. These reciprocal connections between the vestibular nuclei and trigeminal nucleus caudalis may provide a mechanism whereby vestibular signals influence trigeminovascular pathways and trigeminal information processing during migraine attacks [41]. Additionally, studies using functional MRI showed that the vestibular system is represented at a cortical level [42]. The presence of descending cortical projections on vestibular nuclei has been demonstrated in cats. Researchers concluded that neurons in cortical areas were able to modulate vestibular reflexes [43]. Minor cerebellar abnormalities related to eye and arm movements have also been described in asymptomatic migraine patients [20, 35]. Although semicircular canal and otolith afferents terminate in the vestibular nuclei region, both inputs project to the caudal vermis of the cerebellum and Purkinje cells in the cortex of the nodulus/uvula inhibit the vestibular nuclei [44]. These various potential interconnections between migraine and the vestibular system can cause abnormalities in vestibular function tests in migraineurs during the interictal period. A recent blood oxygen level-dependent (BOLD) functional MRI study conducted in patients with VM, patients with migraine without aura, and healthy controls during the interictal period, revealed that caloric vestibular stimulation elicited statistically significant activation in the bilateral insular cortex, thalamus, cerebellum, and brainstem of all subjects [36]. In particular, discrete activation in the periaqueductal gray matter was observed in migraine patients, suggesting a peculiar relationship between vestibular stimulation and the activation of brain areas that play key roles in pain processing [45]. This reciprocal connection between brainstem vestibular nuclei and structures involved in modulation of trigeminal nociceptive inputs may explain the VEMP abnormalities in migraineurs.

Due to the measurement method and/or technical factors, oVEMP is more valuable in assessing vestibular function in patients with headache compared to cVEMP. During cVEMP recordings, amplitude-related parameters change according to the degree of tonic contraction of the SCM showing a direct correlation; the more tonic the muscle tension, the larger the cVEMP amplitude response [4]. Decreased response rate and amplitude or prolonged latencies on cVEMP and oVEMP occur with age increase over 60 years [24, 26]. Regarding the influence of gender on oVEMPs, one study found oVEMPs to be independent of gender [31], whereas another study reported that the mean oVEMP amplitude in males was significantly larger than in females [25]. Thus, in our study, we only included females 20–60 years of age based on these known age and gender effects. To control the amount of muscle tension between right and left muscles, we used a feedback method with a blood pressure manometer and analyzed VEMP parameters following EMG rectification in cVEMP [27, 46]. Many patients with primary headache disorders, such as TTH and migraine, also have accompanying pericranial, neck and shoulder muscle tenderness and/or associated myofascial pain syndrome. These conditions can affect muscle tension or posture during cVEMP measurements. Thus, the cVEMP method may provide inaccurate information in patients with migraine and TTH because the degree of muscle contraction affects the cVEMP result and its interpretation. Consequently, oVEMP may be the more sensitive method for evaluating the vestibular system in primary headache disorders.

Our study had several limitations. First, highly selected patients from a neurology clinic at a regional university hospital were recruited. The sample size was small, and the study used a cross-sectional design that provided limited causal information. Second, the present data did not identify statistically significant correlations between VEMP parameters and headache clinical parameters such as frequency, duration, and intensity (data not shown). Additionally, this study was based on outpatient subjects and only administered the headache questionnaire at the first visit; therefore, detailed headache characteristics recorded using a headache diary should be considered in future studies to more accurately identify the correlations between electrophysiological data and headache parameters. Furthermore, prospective longitudinal studies including information regarding impact or disabilities due to headache may be warranted. Third, sound stimulation was applied at 500 Hz, which showed a 100 % response rate in both oVEMP and cVEMP of healthy subjects [47]. However, VEMPs were not obtained in approximately 8-10 % of subjects in the control group, as previous studies showed similar results [14, 16]. oVEMP and cVEMP predominantly represent saccular stimulation, and bone vibration activates both utricular and saccular afferents [4]. Thus, we should consider the use of a bone vibrator in future studies.

## Conclusions

In conclusion, this study provides electrophysiological evidence that abnormalities of the oVEMP pathway can be observed in patients with migraine without aura who are not experiencing vestibular symptoms during a headache-free period. These findings appear to be related to subclinical vestibulopathy in migraineurs. Thus, oVEMP may be useful in evaluating alterations in the vestibular system in patients with migraine as well as other types of primary headaches.

## Abbreviations

oVEMP: Ocular vestibular-evoked myogenic potential
cVEMP: Cervical vestibular-evoked myogenic potential
ICHD-3β: The International Classification of Headache Disorders-3 beta version
VM: Vestibular migraine
EMG: Electromyography
nHL: Normal hearing level
AR: Amplitude asymmetry ratio
PET: Positron emission tomography
BOLD: Blood oxygen level-dependent imaging
